# Why aren't rabbits and hares larger?

**DOI:** 10.1111/evo.14187

**Published:** 2021-03-11

**Authors:** Susumu Tomiya, Lauren K. Miller

**Affiliations:** ^1^ Center for International Collaboration and Advanced Studies in Primatology Kyoto University Primate Research Institute Inuyama Aichi 484–8506 Japan; ^2^ Negaunee Integrative Research and Gantz Family Collections Centers Field Museum of Natural History Chicago IL 60605 USA; ^3^ Museums of Paleontology and Vertebrate Zoology University of California Berkeley CA 94720 USA; ^4^ Department of Integrative Biology University of California Berkeley CA 94720 USA

**Keywords:** Body size, competition, macroecology, macroevolution, Lagomorpha

## Abstract

Macroevolutionary consequences of competition among large clades have long been sought in patterns of lineage diversification. However, mechanistically clear examples of such effects remain elusive. Here, we postulated that the limited phenotypic diversity and insular gigantism in lagomorphs could be explained at least in part by an evolutionary constraint placed on them by potentially competing ungulate‐type herbivores (UTHs). Our analyses yielded three independent lines of evidence supporting this hypothesis: (1) the minimum UTH body mass is the most influential predictor of the maximum lagomorph body mass in modern ecoregions; (2) the scaling patterns of local‐population energy use suggest universal competitive disadvantage of lagomorphs weighing over approximately 6.3 kg against artiodactyls, closely matching their observed upper size limit in continental settings; and (3) the trajectory of maximum lagomorph body mass in North America from the late Eocene to the Pleistocene (37.5–1.5 million years ago) was best modeled by the body mass ceiling placed by the smallest contemporary perissodactyl or artiodactyl. Body size evolution in lagomorphs has likely been regulated by the forces of competition within the clade, increased predation in open habitats, and importantly, competition from other ungulate‐type herbivores. Our findings suggest conditionally‐coupled dynamics of phenotypic boundaries among multiple clades within an adaptive zone, and highlight the synergy of biotic and abiotic drivers of diversity.

Efforts to unravel the biotic and abiotic drivers of biological diversity can be traced back at least to Lyell's (1832) *Principles of Geology*, which highlighted the deep temporal dimension of the problem and paved the way for the Darwinian revolution in evolutionary biology (cf. Egerton 1968). Almost two centuries later, our constantly expanding knowledge of Earth's past continues to provide fresh insights into the dynamics of biodiversity. In this field of inquiry, there has been a longstanding debate on the relative importance of biotic versus abiotic processes for shaping diversity (e.g., Van Valen 1973; Vrba 1985, 1992; Benton 1987), generating dichotomous views that have come to be called the Red Queen and Court Jester models (Barnosky 2001; Benton 2009; but see Liow et al. 2011 on the less restrictive original Red Queen hypothesis of Van Valen [1973]). Recent studies, however, point to the critical need to better understand the linkage of those processes (Ezard et al. 2011; Liow et al. 2011; Condamine et al. 2019). The present study helps fill that knowledge gap by clarifying the complementary roles of climate, predation, and inter‐clade competition in shaping the evolutionary trajectory of the mammalian order Lagomorpha (pikas, rabbits, and hares). While processes that regulate diversity are typically investigated in clades with strikingly high taxic and phenotypic diversities, it is difficult to disentangle multitudes of forces acting on disparate constituents of such clades. Less speciose and more homogeneous groups such as lagomorphs offer uniquely‐valuable study systems because they bring into focus the factors that limit diversity.

Lagomorphs are, if anything, remarkably successful mammals. The approximately 92 extant species are distributed across all continents except Antarctica, collectively inhabiting a wide range of environments from tropical forests to arctic deserts. In many regions, they are locally abundant and represent a major element of the small‐mammalian herbivore guild (Chapman and Flux 2008; Lacher et al. 2016). Considering such ecological success, the diversity of lagomorphs is strikingly limited both taxically and phenotypically compared to those of rodents (extant sister clade of lagomorphs with ∼2400 living species; Lacher et al. 2016) and terrestrial artiodactyls (another widespread group of herbivorous mammals with ∼380 living species; Wilson and Mittermeier 2011). Importantly, the narrow phenotypic range of wild lagomorphs is not a mere reflection of their limited taxic diversity: their per‐lineage rates of body size evolution have been low compared to those of rodents and artiodactyls (Venditti et al. 2011), and contrary to the evolutionary lability of domestic rabbits under artificial selection (cf. Darwin 1868).

A peculiar aspect of today's low lagomorph diversity is the negative skew of their body size distribution (Fig. 1A; sample‐size adjusted Fisher‐Pearson coefficient of skewness *G*_1_ = −1.1 for body mass data from Jones et al. 2009; see also Gardezi and da Silva [1999]). It is in contrast to the positive skews of rodents (*G*_1_ = 0.90), terrestrial artiodactyls (*G*_1_ = 0.11), and indeed, late Quaternary terrestrial mammals in general (Smith and Lyons 2011), which has been attributed to diffusive body size evolution under the combination of a lower boundary and gradually increasing extinction risk above the modal body mass (Clauset and Erwin 2008). The opposite skew of extant lagomorph body mass distribution therefore suggests the existence of an upper boundary at ∼5 kg in the wild in continental settings. This apparent barrier has been crossed by some domesticated rabbits such as the Flemish Giant rabbit—a breed of *Oryctolagus cuniculus* weighing ∼7−8 kg (Roth and Cornman 1916)—and an extinct leporid from the island of Menorca, *Nuralagus rex*, with the estimated mean body mass of ∼8 kg (Quintana et al. 2011; Moncunill‐Solé et al. 2015). We therefore inferred that the upper body mass boundary in wild continental lagomorphs is set not intrinsically but ecologically by ubiquitous processes (cf. Lomolino et al. 2012).

**Figure 1:**
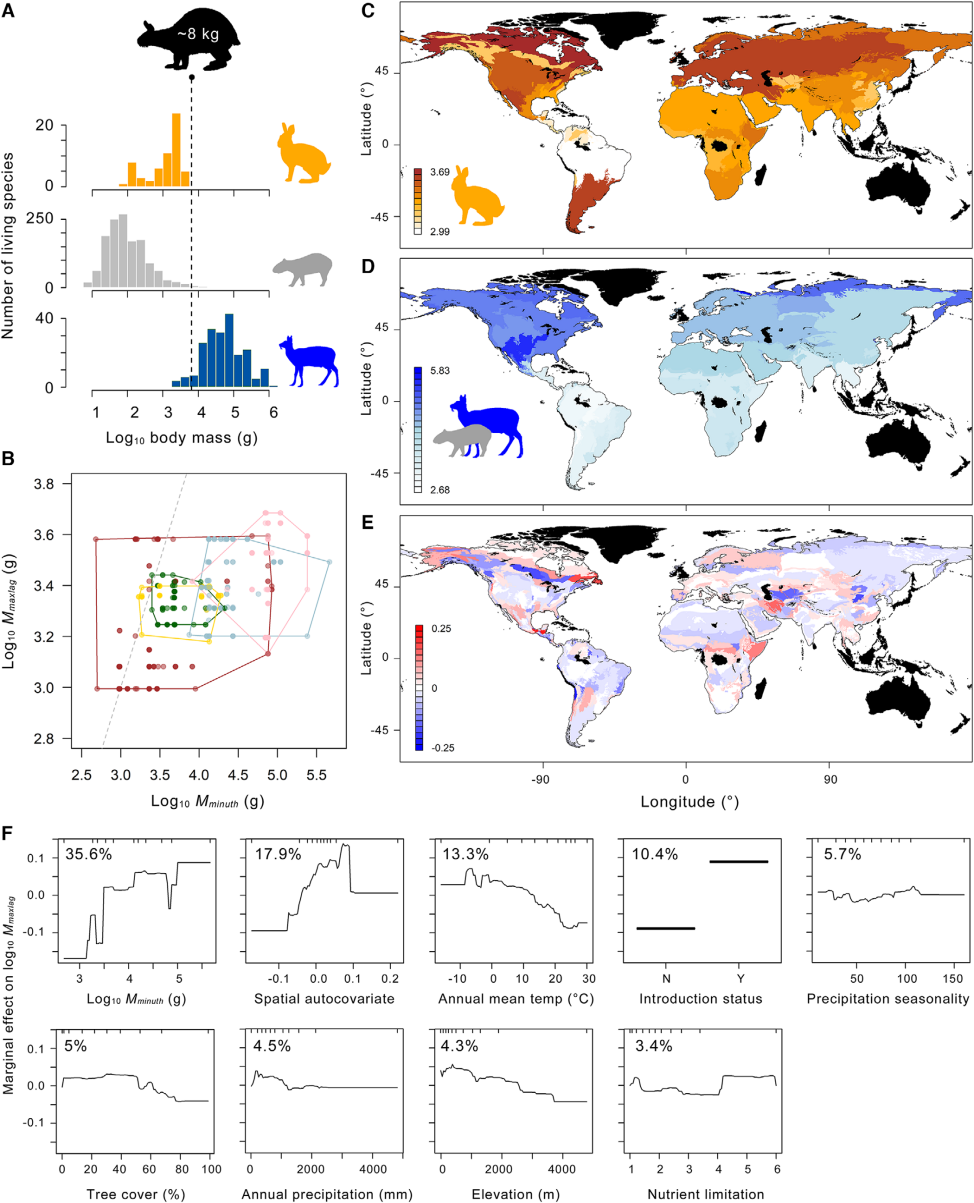
Modern macrogeography of maximum lagomorph body size. (A) Global body mass distributions (data from Jones et al. 2009) in extant lagomorphs (orange), rodents (gray), and artiodactyls (blue); extinct insular leporid *Nuralagus rex* (black) for comparison. (B) Bivariate plot of maximum lagomorph body mass (*M_maxlag_*) and minimum ungulate‐type herbivore body mass (*M_minuth_*; predominantly artiodactyls) in 547 ecoregions in Indo‐Malay (gold), Afrotropical (green), Palearctic (light blue), Nearctic (pink), and Neotropical (brown) realms, against line of equality (gray). (C–E) Log_10_ *M_maxlag_* (C), log_10_ *M_minuth_* (D), and residuals of boosted regression‐tree model predicting log_10_ *M_maxlag_* (E) across ecoregions. (F) Partial dependence plots from boosted regression‐tree analysis of log_10_ *M_maxlag_* in modern ecoregions. Percent values represent relative influences of predictors. Silhouettes from PhyloPic (http://phylopic.org): *N. rex* by Steven Traver, *Cuniculus paca* by Margot Michaud, and *Moschus chrysogaster* by Andrew Farke (https://creativecommons.org/publicdomain/zero/1.0/); Leporidae by Sarah Werning (http://creativecommons.org/licenses/by/3.0/).

In modern ecoregions, terrestrial artiodactyls or ungulate‐like caviomorph rodents frequently occupy the herbivore size class immediately above those of lagomorphs, and play similar ecological roles (Dubost 1968). In fact, lagomorphs have been described as miniature analogs of ungulates (here loosely defined as hoofed mammalian herbivores) with regard to their dietary and locomotor behaviors (Vaughan 1978; Rose 2006). We, therefore, hypothesized that the evolution of maximum average body size in lagomorphs has been competitively constrained by small ungulates and other small ungulate‐like herbivores because lagomorphs lose their competitive advantage above certain body size (or sizes, depending on the environment). Previously, Vaughan (1978), Rose (2006), and Yamada (2017) surmised that competitive pressures from other mammalian groups such as ungulates and rodents may have somehow limited the diversity of lagomorphs. We distilled this idea into quantitative models that focused on body size because it defines a major biological axis along which many physiological, ecological, and life‐history traits strongly covary (Eisenberg 1981; Demment and Van Soest 1985). Competition between species in the same ecological guild is thus expected to intensify with increasing similarity in body size, unless attenuated by niche differentiation (MacArthur and Levins 1967; Gotelli and Graves 1996); conversely, to the extent that body size constraints feeding ecology, size separation tends to enable coexistence of mammalian herbivores that use overlapping resources (Laca et al. 2010).

In this study, we first examined the biogeographic patterns of maximum lagomorph body masses in modern ecoregions and evaluated their potential biotic‐ and abiotic‐environmental predictors including the minimum body masses of co‐occurring ungulate‐type herbivores. We then examined the scaling of local‐population energy use with body mass (Damuth 1981) in extant lagomorphs and ungulates to seek a mechanistic explanation for the observed macrogeographic patterns of body size relationships from an evolutionary‐ecological perspective. Finally, focusing on North America, which long served as the center stage for the evolution of leporids (Dawson 2008; Lopez‐Martinez 2008; Ge et al. 2013), we estimated body masses of fossil lagomorphs within a phylogenetic framework, and evaluated ecological models of the maximum lagomorph body mass since the late Eocene, 37.5 million years ago. These spatial and temporal analyses compensate for the shortcomings of each other: on one hand, the modern geographic distributions and co‐occurrence patterns of species may be heavily affected by human activities (Verde Arregoitia et al. 2015; Lyons et al. 2016); on the other hand, our knowledge of fossil taxa and their paleoenvironment is much more limited.

## Methods

Unless noted otherwise, computations were performed in the R programming environment ver. 3.6.0 (R Development Core Team 2018). Data sets are archived in the Dryad Digital Repository as Electronic Supplementary Materials (ESMs; Tomiya and Miller 2021).

### MODELING OF MAXIMUM LAGOMORPH BODY MASS IN MODERN ECOREGIONS

Based on present range maps (IUCN 2013) and species mean body mass data (Jones et al. 2009), the largest leporid lagomorph and the smallest ungulate‐type herbivore species (excluding lagomorphs) were identified in 574 non‐island ecoregions (Olson et al. 2001; ESM Dataset 1). We considered the following taxa to be ungulate‐type herbivores (UTHs): artiodactyls, perissodactyls, and ungulate‐like or leporid‐like caviomorphs (cf. Dubost 1968; Seckel and Janis 2008) consisting of *Cuniculus*, *Dasyprocta*, *Myoprocta*, *Dolichotis*, *Chinchilla*, *Lagidium*, and *Lagostomus*. Omnivorous or semiaquatic taxa (suids, tayassuids, hippopotamids, and *Hydrochoerus*) were excluded.

We conducted boosted regression‐tree analysis (Elith et al. 2008) of the maximum lagomorph body mass (log_10_ *M_maxlag_*) with the following predictors: (1) minimum UTH body mass (log_10_ *M_minuth_*); (2) mean annual temperature (Hijmans et al. 2005); (3) mean annual precipitation (Hijmans et al. 2005); (4) precipitation variance (as a measure of seasonality) (Hijmans et al. 2005); (5) soil nutrient availability (Fischer et al. 2008); (6) mean tree cover (Hansen et al. 2013; downsampled by averaging to a 30‐minute resolution); (7) elevation (Hijmans et al. 2005); (8) introduction status of the largest lagomorph; and (9) mean residual of neighboring ecoregions as a spatial autocovariate (Crase et al. 2012). Mean predictor values for individual ecoregions were calculated from digital spatial data using the program QGIS (QGIS Development Team 2018) and the R packages ‘rgdal’ version 1.4‐3 (Bivand et al. 2018) and ‘rgeos’ version 0.4‐3 (Bivand and Rundel 2019). The model of *M_maxlag_* was developed using the cross‐validation method (Elith et al. 2008) and the tree complexity, learning rate, and bag fraction values of 3, 0.005, and 0.75, as recommended by Elith et al. (2008). This analysis was performed using the package ‘gbm’ version 2.1.5 (Greenwell et al. 2019) and the script ‘brt.functions.R’ (Elith et al. 2008).

### LOCAL‐POPULATION ENERGY USE

The theoretical scaling of energy use E by a local population of individuals with body mass M was calculated from empirically‐derived allometric scaling patterns of basal metabolic rate (i.e., individual energy use) $R\;=\;aM^{b}$ and local population density $D\;=\;cM^{d}$ as follows (cf. Damuth 1981):

$$E=\mathrm{RD}=\mathrm{ac}M^{\left(b+d\right)},\;\mathrm{or}$$

(1) $$\begin{array}{ccc} \mathrm{lo}g_{10}E & = & \mathrm{lo}g_{10}R+\mathrm{lo}g_{10}D\;=\mathrm{lo}g_{10}a+\mathrm{lo}g_{10}c\\ & & +\;\left(b+d\right)\mathrm{lo}g_{10}M. \end{array}$$

**Figure 2:**
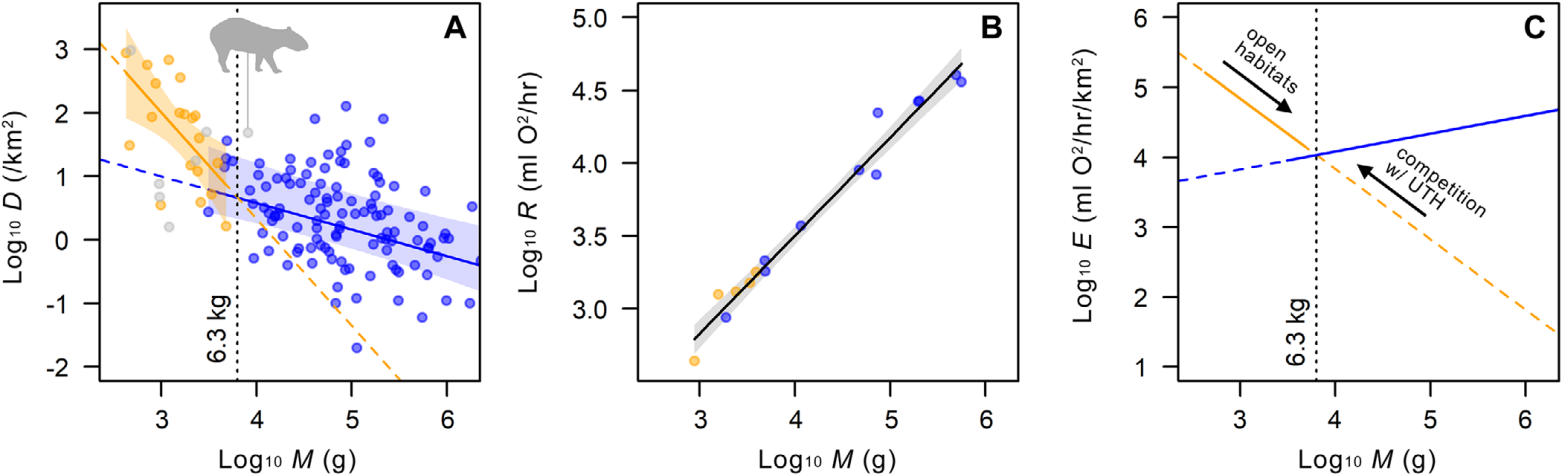
Model of local‐population energy use by extant leporids (orange) and ungulates (blue; herbivorous terrestrial artiodactyls and perissodactyls). Empirical scaling patterns of local‐population density *D* (A) and ‘basal’ metabolic rate *R* (B) against body mass *M*; data for ungulate‐like caviomorphs (gray) are overlaid in (A), with *Cuniculus paca* labeled. (C) Theoretical scaling of local‐population energy use *E* against *M*, illustrating our conceptual model of lagomorph body size evolution: lagomorphs in open habitats experience increased predation pressure and undergo selection for greater cursoriality, resulting in body size increase (right‐pointing arrow); large lagomorphs are kept out of more closed habitats by competitively superior smaller lagomorphs, while further body‐size increase is opposed by the competitive pressure from larger ungulate‐type herbivores (left‐pointing arrow). Polygons represent 95% confidence bands. See Figure 1 caption for PhyloPic credit.

To this end, we performed GLS regression analyses of D and R against body mass (Paradis and Schliep 2019; Smaers and Mongle 2020) using published data for extant leporids, herbivorous terrestrial artiodactyls, and perissodactyls (Jones et al. 2009), and a time‐calibrated species‐level mammalian supertree (Bininda‐Emonds et al. 2007, Bininda‐Emonds et al. 2008); Pagel's (1999) *λ* was constrained to be between 0 and 1 (cf. Revell 2010). Confidence bands for parameter estimates were obtained using the R package ‘evomap’ (Smaers and Mongle 2020). Data on the basal metabolic rates of perissodactyls were not available for the taxa with population density data; we, therefore, assumed that perissodactyls fit the tightly‐constrained scaling of *R* common to leporids and artiodactyls (Fig. 2B). ‘Energy‐equivalent’ lagomorph body masses for observed minimum perissodactyl body masses in the fossil record (see below) were determined by solving Equation (1).

### MODELING OF MAXIMUM LAGOMORPH BODY MASS IN NORTH AMERICAN FOSSIL RECORD

#### Fossil occurrence data

Occurrence data for North American fossil lagomorphs, ungulates (perissodactyls and artiodactyls excluding helohyids, entelodontids, anthracotheriids, and tayassuids), and rodents (to estiamte glires fossil recovery potential; note there are no ungulate‐like terrestrial rodents within the scope of this analysis) were obtained from the Paleobiology Database (Alroy and Uhen 2020, ESM Dataset S5) for the period of 46.2−30.5 million years ago (Ma), and from MIOMAP (Carrasco et al. 2005) and FAUNMAP (Graham and Lundelius 2010) databases for the period of 30.5−0 Ma. Localities (or “collections” [Alroy and Uhen 2020]) and associated taxon occurrence data whose geologic ages had uncertainties exceeding 4.2 million years (equivalent to the duration of Ar2—the longest North American Land Mammal ‘Age’ [NALMA] subage) were excluded. Taxonomic names in the occurrence data set were checked for synonymy and internal consistency (Janis et al. 1998; Prothero and Foss 2007).

The occurrence data were grouped into 1.5 million‐year time bins starting at 43.5 Ma; this temporal resolution was selected in view of the median locality‐age uncertainty of 1.1 million years for the Neogene occurrence data and the median species duration of 2−3 million years for North American fossil mammals (Alroy 2009). We used time bins of equal lengths instead of geochronologic units of uneven durations to simplify the time series analysis. Species were assumed to be present in North America between their first and last appearances. The species‐ and genus‐level taxonomy of fossil lagomorphs follows Dawson (2008). Extant species were assigned body masses from the PanTHERIA database (Jones et al. 2009).

#### Body mass estimation for fossil taxa

Body masses of fossil lagomorphs and ungulates were estimated from published and original measurements of mandibular and dental dimensions (Supplementary Information [SI]; ESM Datasets S2–S4). For lagomorphs, we newly developed allometric models from our measurements of 164 specimens of 34 extant species, for which associated individual body‐mass data were available. Phylogenetic covariance and intraspecific variations were incorporated into the lagomorph models (Garland and Ives 2000; Hansen and Bartoszek 2012; Bartoszek 2019; Paradis and Schliep 2019) using a time‐calibrated molecular tree (Ge et al. 2013). The parameters of the allometric models for fossil ungulates were estimated from a published data set (Mendoza et al. 2006) combined with a time‐calibrated supertree (Bininda‐Emonds et al. 2007, Bininda‐Emonds et al. 2008).

#### Predictor variables

As potential predictors of *M_maxlag_* in North America since the late Eocene, we considered: (1) competitive ceiling body mass, log_10_ *M_ceiling_*, consisting of the energy‐equivalent lagomorph body mass for the contemporary minimum perissodactyl body mass (see Local‐Population Energy Use, above) for the periods of 37.5−24.0 and 15.0−1.5 Ma and the minimum artiodactyl body mass for the period of 24.0−15.0 Ma; (2) minimum perissodactyl body mass, log_10_ *M_minper_*; (3) global benthic δ^18^O (1.5 million‐year means) as a temperature proxy (Zachos et al. 2001); (4) mean North American fossil ungulate hypsodonty index (*H_ung_*) as a precipitation proxy (cf. Fortelius et al. 2002; Eronen et al. 2010); and (5) logit‐transformed range‐through sampling probability for glires genera (i.e., rodents and lagomorphs), *R_glires_*, as a measure of fossil recovery potential for lagomorphs (cf. Tomiya 2013). The mean ungulate hypsodonty index was first calculated for NALMA subages using published data for artiodactyls and perissodactyls (Jardine et al. 2012), and interpolated to the midpoints of the 1.5 million‐year time bins.

#### Model evaluation

After exploratory analyses, we compared the fit of 11 linear regression models with autocovariates to the observed time series of log_10_ *M_maxlag_* from 37.5 to 1.5 Ma based on the sample‐size adjusted Akaike information criterion (AIC_c_; Burnham and Anderson 2002). Five of them (Models 1−5) each included one of the five predictor variables, and the remaining six models each included two predictors as follows: *M_ceiling_* and *R_glires_* (Model 6), *M_minper_* and *R_glires_* (Model 7), *M_ceiling_* and *H_ung_* (Model 8), *M_minper_* and *H_ung_* (Model 9), *M_ceiling_*, and δ^18^O (Model 10), *M_minper_* and δ^18^O (Model 11); given the result, we did not consider models with more than two predictors. The autocovariate for each model was defined as the initial model residual for the preceding time bin, obtained from a preliminary regression analysis that ignored the potential autocorrelation of residuals. For our data set, this approach was more effective at removing autocorrelation than the use of first‐order autoregressive models, based on inspection of autocorrelograms and comparison of AIC_c_ values.

We excluded the most recent 1.5 million‐year interval from our analysis so as to avoid influences of human activities on species associations and body mass distributions (Lyons et al. 2016; Smith et al. 2018). To take into account the uncertainties in the ages of fossil localities and body mass estimates, this analysis was repeated 1,000 times, each time stochastically generating a set of locality ages (from uniform distributions bounded by the maximum and minimum ages of individual localities) and a set of lagomorph and ungulate body mass estimates (from normal distributions with the means equal to the point estimates and variances informed by the models; Garland and Ives 2000; Hansen and Bartoszek 2012). The regression analyses were performed with the R package ‘nlme’ (Pinheiro et al. 2012).

## Results

In all 574 modern continental terrestrial ecoregions analyzed, the smallest ungulate is an artiodactyl, and in 133 of them (131 of 143 Neotropical and 2 of 107 Nearctic ecoregions), the smallest ungulate‐type herbivore excluding leporids (UTH) is a caviomorph rodent. At the ecoregional level, the largest lagomorph and the smallest UTH tend to have similar body masses in parts of the Indo‐Malay and Afrotropical realms, where some of the smallest extant artiodactyls weighing less than 10 kg occur, as well as in Neotropical ecoregions with ungulate‐like caviomorphs (Fig. 1B–D). In Palearctic ecoregions, the two groups are typically separated by a moderately large body mass gap, and in many Nearctic ecoregions, the body mass gap is especially pronounced because small ungulates weighing less than ∼50 kg are generally absent. Overall, the body mass gap widens with increasing minimum UTH body mass (Fig. 1B).

Boosted regression‐tree analysis of the modern pheno‐geographic data generated a model of log_10_ *M_maxlag_* with 5,350 regression trees that explained an average of 76% of the deviance in cross‐validation trials. Of the nine predictors considered here, log_10_ *M_minuth_* was the most influential, accounting for 43% of the contributions of all environmental predictors (excluding the spatial autocovariate; Fig. 1F). Nevertheless, all predictors contributed sufficiently to warrant their inclusion in the final model.

Partial dependence plots (Fig. 1F) show an increase in *M_maxlag_* with increasing *M_minuth_* and decreasing mean annual temperature, and to lesser degrees, with decreasing tree cover (but only down to ∼50%), decreasing mean annual precipitation (but only below ∼1,500 mm/year), decreasing elevation, and highest levels of nutrient limitation. In addition, the largest lagomorph in an ecoregion tended to be larger when it was an introduced species. The relationship between each predictor and the predicted maximum lagomorph body mass is generally better described as polygonal than simply linear (SI Fig. S1), as is often the case in macroecological patterns (Brown 1995; Gaston and Blackburn 2008). Prediction residuals from the global model do not show any major geographic bias at the level of realms, implying that similar rules govern *M_maxlag_* in ecoregions across biogeographic realms (Fig. 1E; SI Fig. S2).

Next, we compared the expected local‐population energy use in extant leporids and ungulates (predominantly herbivorous terrestrial artiodactyls but also including perissodactyls). Our analysis suggests that, while the scaling of metabolic rate with body mass in leporids and ungulates can be adequately described by the same regression line (Fig. 2B), the two groups exhibit markedly different patterns of population density scaling with body mass (Fig. 2A; SI Table S3). Among leporids, the local population density can be very high for small‐sized species, but declines sharply with increasing body mass, and much more so than in ungulates. As a result, the equilibrial body mass in leporids and ungulates with respect to the local‐population energy use occurs at ∼6.3 kg (Fig. 2C), near the observed maximum mean body mass of continental lagomorphs at ∼5 kg. Below this energetically‐equilibrial body mass, leporids are expected to be competitively dominant over similar‐sized ungulates if they share resources. Above it, ungulates of any size should have an advantage over similar‐sized leporids where they co‐occur, unless leporids are somehow able to ‘bend’ the allometric curve upwards by a substantial degree—an evolutionary adjustment that they have apparently been unable to make.

**Figure 3:**
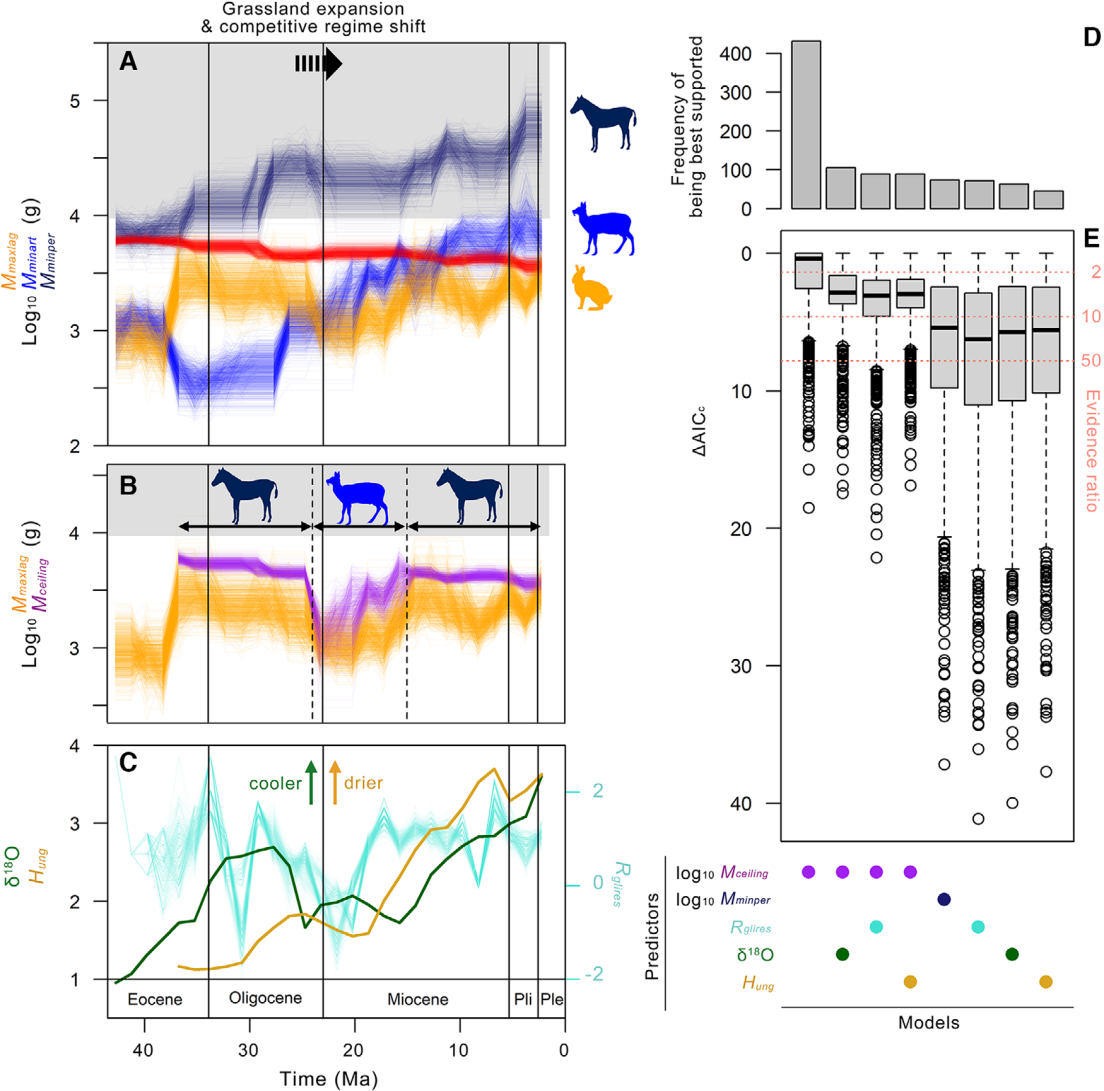
Macroevolution of maximum lagomorph body size in North America. (A) Trajectories of maximum lagomorph body mass (*M_maxlag_*; orange), minimum perissodactyl body mass (*M_minper_*; dark blue), minimum artiodactyl body mass (*M_minart_*; blue), and energy‐equivalent lagomorph body mass for contemporary minimum perissodactyl body mass (red), showing uncertainties in locality ages and body mass estimates across 1,000 pseudo‐replicates; gray area corresponds to body mass range in which near‐complete reliance on rumen fermentation is energetically sustainable (Demment and Van Soest 1985). Lagomorphs in body size region above red line would be competitively inferior to smallest contemporary perissodactyl according to scaling patterns of local‐population energy use (Fig. 2C). (B) Competitive ceiling body mass (*M_ceiling_*; purple), which combines energy‐equivalent lagomorph body mass for contemporary minimum perissodactyl body mass (37.5−24.0, 15.0−1.5 Ma) and minimum artiodactyl body mass (24.0−15.0 Ma) as seen in (A). (C) Additional potential predictors of *M_maxlag_*, including global benthic δ^18^O (green), mean ungulate hypsodonty (*H_ung_*; beige), and range‐through sampling probability for glires genera (*R_glires_*; 1,000 pseudo‐replicates in aquamarine). (D, E) Top eight models (out of 11 compared) of maximum lagomorph body mass for 37.5−1.5 Ma, showing number of times each model received most support (D) and distributions of ΔAIC_c_ for 1000 pseudo‐replicates (E). *Pliohippus* silhouette by Zimices (http://creativecommons.org/licenses/by/3.0/) from PhyloPic (http://phylopic.org); see Figure 1 caption for additional credits.

The estimated mean adult body masses for 74 fossil species of North American lagomorphs from the past 43.5 million years ranged from 0.1 to 2.6 kg (SI Fig. S3; ESM Dataset S3), falling within the range of mean body masses for extant species (0.07−4.80 kg; Fig. 1A). During the entire existence of lagomorphs in that continent, the smallest ungulate has always been an artiodactyl and not a perissodactyl (Fig. 3A; SI Fig. S3). The oldest lagomorphs in North America (*Mytonolagus* spp.) are estimated to have weighed 0.8 kg, comparable to small‐ to medium‐sized extant species of North American cottontail rabbits (*Sylvilagus* spp.). After first appearing at ∼44−43 Ma (Dawson 2008), the maximum body size of lagomorphs increased to 2.6 kg (*Megalagus brachydon*) by 37 Ma (Fig. 3A), that is, within the first one‐sixth of the group's history in that continent. The maximum lagomorph body size remained stable for the subsequent ∼12 million years until the late Oligocene. This same period saw abundance of small artiodactyls with estimated body masses on the order of 0.1−1 kg, including some of the smallest known artiodactyls of all time. As a result, the body mass ranges of lagomorphs and artiodactyls overlapped from the late Eocene to the Oligocene (Fig. 3A; SI Fig. S4A).

The transition from the Oligocene to the Miocene Epoch was marked by extinctions of very small artiodactyls and hare‐sized (>2 kg) lagomorphs. Consequently, the body size ranges of lagomorphs and artiodactyls rapidly segregated, and their opposing body mass boundaries gradually increased thereafter, closely tracking each other from the late Oligocene until the middle Miocene, ca. 24−15 Ma (Fig. 3A). Beginning in the latter half of the Miocene, the rise in the maximum lagomorph body mass appears to have slowed down or hit a ceiling, increasingly lagging behind the minimum artiodactyl body mass and widening the size gap between the two groups (SI Fig. S4A). This trend parallels the tropical‐to‐Holarctic realm‐level transition in modern ecoregions (SI Fig. S5). Still, for most of the past 43.5 million years, the smallest artiodactyls remained smaller than: (1) the energetically equilibrial body mass of ∼6.3 kg (Figs. 2C and 3A), which would have put them at a competitive disadvantage against similar‐sized lagomorphs if their resource uses had largely overlapped; and (2) the ∼9.4 kg threshold for transition to a digestive system that is heavily reliant on foregut fermentation (Demment and Van Soest 1985; gray areas in Fig. 3A and B).

Unlike the artiodactyls, the smallest perissodactyls—the likes of which are absent in modern faunas—maintained substantially larger body sizes (generally by an order of magnitude or more) than contemporary lagomorphs throughout their history in North America (Fig. 3A; SI Fig. S4B). However, from the late Eocene to the late Oligocene and again from the middle Miocene onwards, the maximum lagomorph body mass closely tracked the theoretical energy‐equivalent lagomorph body mass for the smallest perissodactyl in the same time bin (red lines in Fig. 3A; derived from the scaling patterns of local‐population energy use in Fig. 2C). The latter remained relatively stable over the entire history of coexistence of lagomorphs and perissodactyls in the continent despite an approximately eightfold increase in the minimum perissodactyl body mass between 43.5 and 1.5 Ma—a consequence of the much shallower slope of local‐population energy use against body mass in ungulates compared to lagomorphs (Fig. 2C).

From these historical patterns, we visually identified a set of apparent body mass ceilings placed on lagomorphs by perissodactyls (37.5−24.0 and 15.0−1.5 Ma) and artiodactyls (24.0−15.0 Ma), here termed the ‘competitive ceiling body mass’ (*M_ceiling_*), as a potentially powerful predictor of the maximum lagomorph body mass in the North American fossil record (purple lines in Fig. 3B). In essence, it represents a series of the smaller of (A) the energy‐equivalent lagomorph body mass for the minimum perissodactyl body mass (red lines in Fig. 3A) and (B) the minimum artiodactyl body mass (blue lines in Fig. 3A), which must also be larger than the maximum lagomorph body mass (hence the “ceiling”). Information‐theoretic comparison of regression models (Fig. 3D, E) showed that taking into consideration the uncertainties in locality ages and body mass estimates, the maximum lagomorph body mass between 37.5 Ma and 1.5 Ma was indeed best predicted by (and positively correlated with) the ‘competitive ceiling body mass’ rather than by the minimum perissodactyl body mass, global benthic δ^18^O value (a global temperature proxy; Zachos et al. 2001), mean hypsodonty index for North American ungulates (*H_ung_*, a precipitation proxy; Eronen et al. 2010), or range‐through sampling probability for glires genera (*R_glires_*, a measure of fossil recovery potential). Moreover, the next three best models (generally with evidence ratios of <10) all included *M_ceiling_* as one of the predictors, in combination with δ^18^O, *R_glires_*, or *H_ung_*.

## Discussion

Macroevolutionary consequences of competition have long been investigated in divergent patterns of lineage diversification among speciose clades (e.g., Van Valen and Sloan 1966; Gould and Calloway 1980; Stanley and Newman 1980; Cifelli 1981; Krause 1986; Maas et al. 1988; Janis 1989; Benton 1996; Sepkoski Jr 1996; Van Valkenburgh 1999; Rabosky 2013; Pedersen et al. 2014; Liow et al. 2015; Silvestro et al. 2015; Condamine et al. 2019). However, even when inverse diversity dynamics are observed among higher taxa (i.e., the rise of one group is accompanied by the fall of another), the underlying mechanism is rarely clear from analysis of taxon counts alone (Jablonski 2008; Liow et al. 2015). Moreover, there is growing evidence that speciation and phenotypic evolution in mammals are not as tightly coupled as traditionally assumed (Venditti et al. 2011; Slater 2015), such that exclusive focus on taxic diversity may miss important processes that shape biological diversity. Additional insights have come from studies tracking phenotypic ranges of potentially‐competing lineages in the fossil record (e.g., Janis et al. 1994; Hopkins 2007; Brusatte et al. 2008; Friscia and Van Valkenburgh 2010; Kimura et al. 2013; Benson et al. 2014; Slater 2015). Lagomorphs and other ungulate‐type herbivores together present a relatively simple—and thus ideal—study system for interpreting the dynamics of phenotypic boundaries because: (1) they are extant clades with extensive fossil records; (2) their basic ecological adaptations have remained relatively stable through much of their evolutionary histories (Wood 1940; Dawson 2008); (3) as primary consumers, their occurrences are tied directly to vegetation and closely to climate (Leach et al. 2015a; Vrba 1992; Eronen et al. 2010); and (4) the potential for inter‐clade competition is high given their broadly similar dietary and locomotor specializations (cf. Cope 1884; Gidley 1912; Wood 1957; Vaughan 1978; Rose 2006).

The limited body size range of North American fossil lagomorphs reinforces the long‐held perception of evolutionary stability in lagomorphs (Wood 1940; Dawson 2008). Over the past 43.5 million years, their maximum average body mass never exceeded the presently observed upper limit of ∼5 kg in North America (Fig. 3A), and while the history of the crown‐group Lagomorpha likely goes back by an additional 10 million years to ∼53 million years ago (Rose et al. 2008), we are not aware of any fossil lagomorph from any continent that would have weighed much more than 5 kg. At the same time, the trajectory of maximum lagomorph body mass during the Eocene (Fig. 2A, B) illustrates the possibility of rapid body size evolution in lagomorphs as is also suggested by the gigantism of certain domestic breeds and extinct insular species (Roth and Cornman 1916; Quintana et al. 2011; Lomolino et al. 2013; Moncunill‐Solé et al. 2015).

Our analyses of the geographic and historical distributions of lagomorph body masses consistently show that the maximum lagomorph body mass is strongly linked to the minimum body mass of ungulates at relatively large spatiotemporal scales, and suggest that the evolution of lagomorph body size has been constrained by ungulates. This view comes into sharper focus when the local‐population energy use E is considered. First, the distinct scaling patterns of E in extant leporids and ungulates generate the following basic expectations where they compete for limited resources (Fig. 2C; see also Van Valen 1973; Damuth 1981, 1987, 2007): (1) lagomorphs weighing more than ∼6.3 kg are at a competitive disadvantage to, and unlikely to stably coexist with, ungulates of all sizes (left‐pointing arrow in Fig. 2C); (2) large, hare‐type lagomorphs are at a competitive disadvantage to, and unlikely to stably coexist with, smaller, rabbit‐type lagomorphs. Competitive pressure could be mitigated by dietary differentiation, but the combination of morphological, digestive, and behavioral specializations in lagomorphs (Hirakawa 2001; Hume 2002) is apparently not amenable to major departures from a primarily folivorous diet (e.g., frugivory as seen in modern tragulids), making it difficult for lagomorphs to escape intra‐ and inter‐clade competition (Leach et al. 2015b; Hulbert and Andersen 2001).

Expectation 1 is supported by the universal absence of continental lagomorphs weighing much more than ∼6.3 kg where ungulates co‐occur. The close correspondence of the observed continental maximum lagomorph body mass (∼5 kg) with the intersection of the regression lines representing the scaling of *E* in lagomorphs and ungulates (Fig. 2C) is not a mathematical necessity, and cannot be easily explained without invoking evolutionary‐ecological stability (cf. Damuth 1981, 2007). It is noteworthy that ungulate‐like caviomorph rodents, despite belonging to the extant sister clade of lagomorphs, are not confined within the same upper size limit as lagomorphs, and that they may have achieved large body sizes by maintaining relatively high population densities, as in the case of the lowland paca (*Cuniculus paca*; Fig. 2A).

Expectation 2 is supported by the habitat‐level segregation of sympatric small and large leporids into closed and open habitats, respectively (MacCracken 1982; Flux 2008). This phenomenon may be traced back to the late Eocene (Webb 1977), when the largest lagomorphs attained the body masses comparable to those of extant hares (Fig. 3A). Because maximum running speed is correlated with body size (Garland 1983), we interpret the association of large lagomorphs with open grassland habitats as primarily a consequence of elevated predation risks (Flux 2008) and selection for increased cursoriality (right‐pointing arrow in Fig. 2C). At the same time, the steep decline of the local‐population energy use with increasing body mass and the resulting competitively‐induced restriction on the range of environments where large lagomorphs can thrive may have contributed to the generally slow per‐lineage rates of body size evolution in lagomorphs (Venditti et al. 2011).

The scaling relationship of local‐population energy uses in lagomorphs and ungulates allows for estimation of energetically‐equivalent (at the population level) body masses in the two groups. When the minimum perissodactyl body masses in the North American fossil record were converted into energetically‐equivalent lagomorph body masses, much of the apparent body size gap between contemporary lagomorphs and perissodactyls disappeared (dark blue vs. red lines in Fig. 3A). Because lagomorphs exceeding these ecological threshold body masses (red lines in Fig. 3A) are expected to be competitively inferior to the smallest perissodactyls (Fig. 2C), the tight body mass ceiling placed above the lagomorphs by the perissodactyls (purple lines in Fig. 3B) is interpreted to be an evolutionary constraint for the lagomorphs. As pointed out by one of the reviewers of this paper (see also Illius and Gordon 1992), the fact that lagomorphs and perissodactyls are both hindgut fermenters may have intensified the resource competition between the two groups.

Small artiodactyls also played a key role in constraining the body size evolution of lagomorphs, as demonstrated by the model with the competitive ceiling body mass, and especially during the period from the late Oligocene to the middle Miocene (Fig. 3A, B). However, unlike in the case of perissodactyls, the smallest artiodactyls during this time were within the body size range where they are expected to have been at a competitive disadvantage against similar‐sized lagomorphs (i.e., below ∼6.3 kg in Fig. 2C). In that context, the tightly coupled upward shifts of the minimum artiodactyl and maximum lagomorph body masses between ca. 24 and 15 Ma can be interpreted as gradual displacement of artiodactyls by lagomorphs, and the competitive pressure appears to have only slowed down, rather than prevented, the size increase in lagomorphs.

The transition from the perissodactyl‐dominated to artiodactyl‐dominated constraint on lagomorphs approximately coincided with rapid expansion of grassland biomes in midcontinental North America (cf. Strömberg 2005). The resulting restructuring of the competitive regimes for mammalian herbivores evidently reset the course of evolution for lagomorphs, and may have been part of a threshold‐induced critical transition in the large‐scale ecosystem (Scheffer et al. 2012). The second transition back to the perissodactyl‐dominated constraint was roughly contemporaneous with the end of the middle Miocene Climatic Optimum at ∼15 million years ago (Holbourn et al. 2014) and the onset of a trend toward widespread aridification (Eronen et al. 2012). Thus, climate change has likely played major roles in lagomorph evolution, but more as a catalyst of state shifts than a constant driver of phenotypic evolution (see also Barnosky 2005; Figueirido et al. 2012; Orcutt and Hopkins 2013). Although evolutionary response to climate change has been a subject of intense study (see Blois and Hadly [2009] for a review), the modulating role of climate in inter‐clade competition may be a more prevalent feature of evolution than is generally realized (cf. Barnosky 2001; Liow et al. 2011).

More broadly, the persistence of dynamic upper body‐size limits in North American fossil lagomorphs is also consistent with the competitive suppression hypothesis given the ∼12 million years of evolutionary ‘head starts’ that artiodactyls and perissodactyls had over lagomorphs in North America (Rose 2006; Theodor et al. 2007; Dawson 2008). In fact, lagomorphs coexisted with artiodactyls for their entire evolutionary histories in all continents, and in most continents, artiodactyls were already well established when lagomorphs first appeared there (Lopez‐Martinez 2008; Winkler and Avery 2010; Flynn et al. 2014). Moreover, the same sequence applies to lagomorphs and caviomorph rodents in South America (Woodburne 2010; Vucetich et al. 2015). These temporal patterns conform to the phenomenon of incumbent advantage, or priority effect, at a macroevolutionary scale, which has been observed in a wide array of vertebrate and invertebrate groups (Van Valkenburgh 1999; Valentine et al. 2008; Schueth et al. 2015; Roopnarine et al. 2019). From this historical viewpoint, the positive association between the introduction status and body mass of the largest lagomorphs in modern ecoregions (Fig. 1F), which is mainly observed in the Neotropical realm, may be interpreted as a sign of major anthropogenic defaunation of native ungulates—and thus reduction in competitive pressures—where leporids have been introduced (cf. Dirzo et al. 2014).

It is interesting that the smallest living ungulates such as chevrotains (Tragulidae) and miniature antelopes (Bovidae) are able to coexist with similar‐sized and sometimes even larger leporids at the ecoregional scale (Fig. 1B) in spite of their apparent competitive disadvantage (Fig. 2C). A similar phenomenon of overlapping body size ranges is observed between artiodactyls and lagomorphs in the late Eocene to the late Oligocene of North America (Fig. 3A). These instances of coexistence are likely enabled by dietary separation, with the small ungulates tending more toward frugivory or browsing (Dubost 1984; Gagnon and Chew 2000; Meijaard 2011) and the leporids having the capacity to be more graminivorous (Ge et al. 2013). The importance of such dietary separation is also suggested by the biogeographic history of lagomorphs: in Africa, where tragulids with primarily folivorous rather than frugivorous diet appeared by the early Miocene (Ungar et al. 2012), the establishment of large, leporid‐type lagomorphs was much delayed until the Quaternary Period despite: (1) the presence of the order there since the early Miocene (Winkler and Avery 2010) and (2) the rapid spread of leporids across the neighboring continent of Eurasia after ∼8 Ma (Flynn et al. 2014).

Among ruminants, heavy utilization of high‐quality foods such as fruits is most feasible in small‐bodied taxa in tropical to subtropical forests (Demment and Van Soest 1985; Fleming et al. 1987). Such environments are rare in the Palearctic and Nearctic ecoregions, resulting in the frequent absence of very small ungulates and contributing to the generally greater body‐size separation of the two groups (Fig. 1B; SI Fig. S5). The same explanation fits the upward shift in the minimum body mass of ungulate‐type herbivores in the late Miocene of North America (Fig. 3A; SI Fig. S4), which coincided with the spread of arid biomes across the continent (Eronen et al. 2012). Nevertheless, we caution against inferring local paleoenvironments from the body‐size relationship of the two groups alone: as a case in point, the lagomorph and artiodactyl body‐mass ranges overlapped substantially in the late Eocene to the late Oligocene of North America (Fig. 3A), when the most fossiliferous midcontinental regions were probably cooler and drier than today's tropics (Retallack 2007; Zanazzi et al. 2007; Boardman and Secord 2013; Eronen et al. 2015; Pound and Salzmann 2017). Additional research into the ecology of herbivorous mammals during this peculiar chapter of mammalian evolution is warranted.

## Conclusion

We found remarkably broad concordance between the patterns of the modern pheno‐geography and the paleontological time series, both pointing to prevalent evolutionary constraints placed on lagomorphs by ungulate‐type herbivores. From the mechanistic standpoint, lagomorphs offer perhaps the clearest evidence yet for the significant role of competition in dynamic subdivision of an adaptive zone (cf. Van Valen 1973; Liow et al. 2011), bridging tiers of evolutionary time (cf. Gould 1985). Our findings also provide empirical support for Damuth's (1981, 1987, 2007) energy‐equivalence rule as a powerful guiding principle for interpreting the history of biological diversity.

## AUTHOR CONTRIBUTIONS

S.T. conceived of the study, collected data, carried out analyses, drafted the manuscript, and coordinated the study. L.K.M. collected data and improved data collecting procedure. Both authors gave final approval for publication.

## DATA ARCHIVING

The Electronic Supplementary Materials, including lists of museum specimens and their repositories, are archived in the Dryad Digital Repository (https://doi.org/10.5061/dryad.ns1rn8ps3).

Associate editor: A. Goswami

Handling Editor: D. W. Hall

## Supporting information


**Fig. S1**. Maximum lagomorph body masses in modern ecoregions predicted by boosted‐regression tree model, plotted against individual predictors.
**Fig. S2**. Residuals from boosted regression‐tree analysis of maximum lagomorph body mass (log_10_ *M_maxlag_*) in modern ecoregions, compared across biogeographic realms.
**Fig. S3**. Observed species temporal ranges (horizontal lines and dots [for single occurrences] based on locality midpoint ages) of leporid‐like stem lagomorphs and leporids (orange), ochotonids (pink), artiodactyls (blue), and perissodactyls (dark blue) in North America from 43.5 to 1.5 Ma plotted against body masses (point estimates).
**Fig. S4**. Trajectories of body mass difference between minimum artiodactyl (A) or perissodactyl (B) and maximum lagomorph in North America from 43.5 to 1.5 Ma.
**Fig. S5**. Bivariate plot of maximum lagomorph and minimum artiodactyl body masses in 547 modern ecoregions.
**Fig. S6**. Illustration of dental predictor variables used in lagomorph body‐mass estimation (*Lepus townsendii* [MVZ 105670] as an example).
**Table S1**. Parameter estimates for lagomorph body‐mass prediction models (in order of decreasing predictive accuracy as measured by |*D*|).
**Table S2**. Parameter estimates for ungulate body‐mass prediction models (in order of decreasing predictive accuracy).
**Table S3**. GLS model parameters for population density *D* and ‘basal’ metabolic rate *R* against body mass in extant leporids and ungulates (non‐‘suoid’ artiodactyls and perissodactyls).
