# Supplementary material for: Why aren't rabbits and hares larger?
Source: Evolution. 2021 Mar 11;75(4):847–60. doi: 10.1111/evo.14187 (PMC8252017; doi:10.1111/evo.14187)
Supplement: Supplementary file 1 — Fig. S1. Maximum lagomorph body masses in modern ecoregions predicted by boosted‐regression tree model, plotted against individual predictors. Fig. S2. Residuals from boosted regression‐tree analysis of maximum lagomorph body mass (log10 Mmaxlag) in modern ecoregions, compared across biogeographic realms. Fig. S3. Observed species temporal ranges (horizontal lines and dots [for single occurrences] based on locality midpoint ages) of leporid‐like stem lagomorphs and leporids (orange), ochotonids (pink), artiodactyls (blue), and perissodactyls (dark blue) in North America from 43.5 to 1.5 Ma plotted against body masses (point estimates). Fig. S4. Trajectories of body mass difference between minimum artiodactyl (A) or perissodactyl (B) and maximum lagomorph in North America from 43.5 to 1.5 Ma. Fig. S5. Bivariate plot of maximum lagomorph and minimum artiodactyl body masses in 547 modern ecoregions. Fig. S6. Illustration of dental predictor variables used in lagomorph body‐mass estimation (Lepus townsendii [MVZ 105670] as an example). Table S1. Parameter estimates for lagomorph body‐mass prediction models (in order of decreasing predictive accuracy as measured by |D|). Table S2. Parameter estimates for ungulate body‐mass prediction models (in order of decreasing predictive accuracy). Table S3. GLS model parameters for population density D and ‘basal’ metabolic rate R against body mass in extant leporids and ungulates (non‐‘suoid’ artiodactyls and perissodactyls). [file EVO-75-847-s001.pdf]

# Supplementary Information for: Tomiya, S., and L. K. Miller (2021) Why aren’t rabbits and hared larger? *Evolution*. https://doi.org/10.1111/evo.14187

## Additional Notes on Methods and Results

### *Body Mass Estimation for Fossil Lagomorphs*

**General Approach.** Although allometric equations for predicting body masses of lagomorphs from their cranial, postcranial, and lower first-molar (m1) dimensions exist (Moncunill-Solé et al. 2015), here we newly developed phylogenetically-informed predictive models (Garland and Ives 2000) that: (*i*) are based on skeletal specimens with associated individual body-mass or total-length data (instead of using species-level statistics in the literature that are not directly tied to measured specimens); (*ii*) are applicable to maxillary as well as mandibular tooth measurements and diastemal measurements (so as to maximize their applicability to fossil taxa that are known from fragmentary materials); (*iii*) take into account potential phylogenetic covariances of the model residuals (Revell 2010); (*iv*) are evaluated against a range of trait evolution patterns by incorporating Pagel’s $\lambda$ parameter (a measure of phylogenetic inertia; Pagel 1999); and (*v*) take into account within-species sampling errors in the predictor variables (Hansen and Bartoszek 2012).

**Extant Reference Data.** Dental and diastemal dimensions of 164 skeletal specimens representing 34 extant species in two lagomorph families (Ochotonidae and Leporidae) were measured using digital calipers and ocular micrometers (Electronic Supplementary Material [ESM] Dataset S2, Tomiya and Miller 2021). Each of these specimens had its individual body mass (M) or total length (TL) recorded on a specimen tag. We measured the anteroposterior lengths of: the upper cheek tooth row across alveoli (UARL), lower cheek tooth row across alveoli (LARL), lower diastema (DL), upper and lower first molars (M1L and m1L), and lower third premolar (p3L) as potential predictors of body mass (see Fig. S6 for illustration).

The individual teeth were selected because M1 and m1 are commonly used in body mass estimation of fossil mammals (Legendre 1986; Alroy 1998), and p3 is frequently a key tooth in the taxonomy and morphological diagnosis of lagomorphs (White 1988; Dawson 2008). For USNM specimens, individual teeth could not be measured because ocular micrometer was not available in the collection. Only adult skeletons were measured, and for each species, attempts were made to collect data from (*i*) similar numbers of males and females and (*ii*) a wide range of body sizes.

We adopted a time-calibrated molecular tree of extant lagomorphs (Ge et al. 2013), which included the 34 extant species measured here, as the phylogenetic backbone of the predictive

models. All predictor values were $\log_{10}$-transformed for the analysis to improve the normality of their distributions.

Specimen Repositories: **CAS**, California Academy of Sciences (San Francisco, USA); **FMNH**, Mammal Collection, Field Museum of Natural History (Chicago, USA); **MVZ**, Museum of Vertebrate Zoology, University of California, Berkeley (Berkeley, USA); **USNM**, Division of Mammals, US National Museum of Natural History (Washington, D.C., USA).

**Fossil Data.** Dental and diastemal measurement data for fossil lagomorphs were compiled from published sources (ESM Dataset S3). We conservatively assigned the maximum value of the sample-size-weighted within-species sample variance of body mass ($\sigma_{wiM}^2$; Hansen and Bartoszek 2012) in the extant reference data set to the fossil species.

Genus- and species-level taxonomy followed Dawson (2008). For the purpose of present study, fossil species of the leporid *Sylvilagus* were connected directly to the node on the tree corresponding to the most-recent common ancestor of the extant species of that genus and its closest extant genera (*Pentalagus*, *Bunolagus*, and *Romerolagus*), effectively assuming the monophyly of the genus *Sylvilagus* including the fossil species. All other fossil taxa, whose phylogenetic positions are highly uncertain, were connected directly to the root of the crown Lagomorpha. Some of these taxa are traditionally classified in the family Leporidae (Dawson 2008) but may, in fact, be stem lagomorphs (Rose et al. 2008). Branch lengths for the fossil species were determined based on their first-appearance dates reported by Dawson (2008). The phylogenetic information was edited in the NEXUS file format using the program Mesquite ver. 3.6 (Maddison and Maddison 2018).

**Estimation Procedure.** All computations were performed in the R programming environment ver. 3.5.2 (R Development Core Team 2018). First, mean body masses of 8 extant species, for which individual total lengths (TL), but not body masses (M), were recorded, were estimated from their mean TL using a predictive model (see below) and associated M–TL data for 26 extant species, represented by 145 specimens. This was done to maximize the sample of reference taxa, and we confirmed that body mass and total length were tightly correlated at the interspecific scale ($R^2$ = 0.992 for $\widehat{\lambda_{\mathrm{ML}}} = 0$). The set of mean body masses (observed for 26 species and estimated for 8 species) and corresponding mean dentognathic measurements were then used to derive models for predicting body masses of the fossil species.

Body-mass prediction models (Garland and Ives 2000) were derived as follows: For each predictor variable, generalized least-squares (GLS) regression analysis accounting for phylogenetic relationships and within-species sampling errors (i.e., observational errors, or 'measurement' errors) was performed using the R packages *GLSME* ver. 1.0.5 (Bartoszek 2019), *ape* ver. 5.0 (Paradis and Schliep 2019) and *nlme* ver. 3.1-137 (Pinheiro et al. 2017).

Observational variances were estimated as the average within-species sample variances weighted by sample sizes ($\sigma_{wi}^2$; Hansen and Bartoszek 2012). Because estimates of the response variance constant ($\sigma_y^2$) by the function *GLSME* were found to be highly unstable, we used the function *optimize* of the R *stats* package (R Development Core Team 2018) in combination with the *GLSME* function to obtain maximum-likelihood estimates of $\sigma^2$, and selected the value of Pagel's $\lambda$ parameter (in the variance-covariance matrix for the model residual error term) between 0 (no phylogenetic inertia) and 1 (equivalent to the Brownian-motion model) that maximized the log likelihood of the model. In all cases, corrections for biases in parameter estimates resulting from within-species sampling errors were accepted based on the reliability ratio *K* (Hansen and Bartoszek 2012). Model parameter estimates are provided in Table S1.

Accuracies of body-mass prediction models are typically quantified without utilizing all available phylogenetic information. To compare the performances of models with different predictors, we instead calculated the mean absolute deviation $\overline{|D|}$ of phylogenetically-informed body mass estimates for the 34 extant reference species from their observed body masses, using the leave-one-out procedure (we avoided antilog transformation of the body mass estimates as it would introduce unnecessary bias [Smith 1993]). The predictors in order of decreasing prediction accuracy were: DL, M1L, m1L, LARL, UARL, and p3L (Table S1). The poor performance of the model with p3L is likely attributable to the substantial shifts in the occlusal surface dimensions of lagomorph p3 with tooth wear (Kelly 2000). It may also reflect the greater evolutionary lability of this tooth compared to other cheek teeth, which is evident in the heavy reliance of lagomorph taxonomy on p3 morphology (White 1988, 1991; Dawson 2008).

For each fossil species, the body-mass estimate based on the available predictor with the highest prediction accuracy was adopted; as a result, UARL and LARL were ultimately not used in lagomorph body-mass estimation. Any occurrence of a fossil genus lacking the species-level identification was assigned the mean estimated body mass for the North American species (both fossil and extant, if applicable) of that genus. Body-mass prediction followed the GLS approach of Garland and Ives (2000), and the variance of each body mass estimate was calculated as in Martins and Hansen (1997: eq. 12). All estimates are reported in ESM Dataset S3.

### *Body Mass Estimation for Fossil Ungulates*

**Extant Reference Data.** Species-mean dental dimensions for extant artiodactyls (excluding suids, tayassuids, and hippopotamids) and perissodactyls were extracted from the data set of Mendoza et al. (2006). As potential predictors of body mass, we analyzed the lower molar-row length (LMRL) and the anteroposterior lengths (L) and labiolingual widths (W) of the upper second (M2) and the lower first through third molars (m1–m3). The time-calibrated supertree of Bininda-Emonds et al. (2007, 2008) was adopted as the phylogenetic backbone of the GLS analysis and body-mass prediction.

**Fossil Data.** Tooth-size data for North American fossil species of artiodactyls and perissodactyls were downloaded from the Paleobiology Database (www.fossilworks.org) on March 8–9, 2020, and were supplemented by our original measurement data (ESM Dataset S4). Taxonomic names were checked for synonymy against the fossil occurrence data. The fossil taxa were grafted to the tree of extant ungulates based generally on their taxonomic affiliations at the level of family or above; thus, as in the case of fossil lagomorphs, we took a statistically conservative approach (Garland and Ives 2000) and avoided making precise phylogenetic assumptions. Branch lengths leading to the fossil species were calculated based on their first-appearance dates (the earliest midpoint ages of localities that have yielded those species) in our taxon occurrence data set (see Methods of main text); this procedure led to a slightly negative branch length in one case (*Pseudoblastomeryx advena*), which we then forced to be zero.

**Estimation Procedure.** The body-mass prediction followed the same procedure as for the fossil lagomorphs, except that observational ('measurement') errors could not be incorporated into the model building process in the absence of information on intraspecific size variations in the adopted data set (Mendoza et al. 2006). Although that may have led to biased body-mass estimates for the fossil ungulates (Hansen and Bartoszek 2012), we have no reason to suspect that it affected subsequent analyses qualitatively. The predictors in order of decreasing prediction accuracy were: LMRL, m2L, m1L, M2L, m1W, M2W, m2W, m3W, and m3L (Table S2). Fossil species for which measurement data corresponding to these predictors were unavailable were assigned the mean estimated body masses of the genera to which they belonged (ESM Dataset S4).

### ***Local-Population Energy Use***

Taxonomic names were standardized between the trait data and the phylogenetic data by referring to the IUCN Red List (IUCN 2013). *Alcelaphus caama*, *A. lichtensteinii*, *Beatragus hunteri*, *Damaliscus korrigum*, and *Oryx beisa* were excluded from this analysis because they could not be placed on the adopted supertree. *Capreolus capreolus* was excluded from the regression analysis of metabolic rate after it was identified as an outlier by an initial analysis. We note that truly basal metabolic rates may not be readily measurable in herbivores that employ high levels of fore- or hindgut fermentation; thus, the parameter estimates for the scaling of metabolic rate reported here may not be directly comparable to those for other mammalian clades (Capellini et al. 2010).

**Fig. S1**. Maximum lagomorph body masses in modern ecoregions predicted by boosted-regression tree model, plotted against individual predictors.

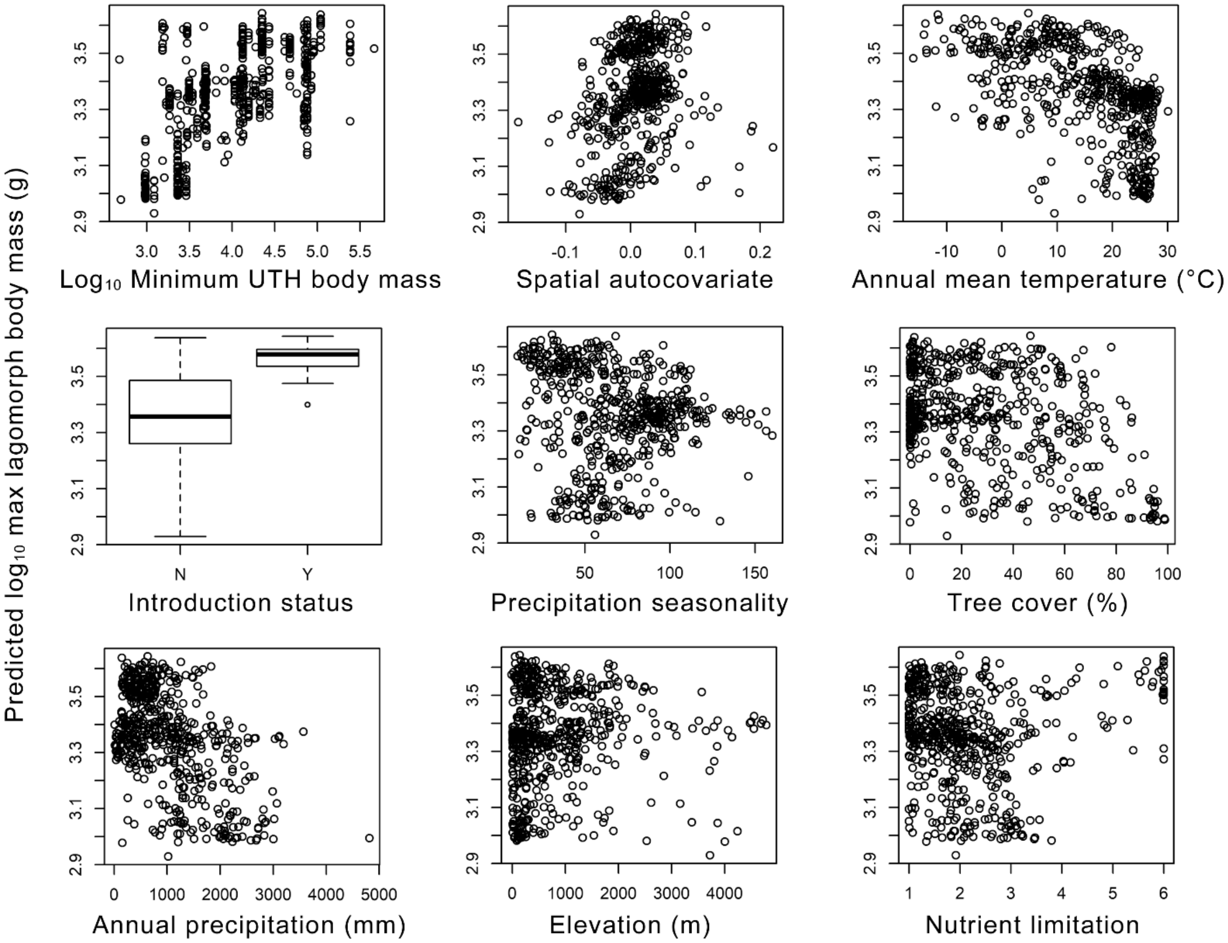

**Fig. S2.** Residuals from boosted regression-tree analysis of maximum lagomorph body mass ($\log_{10} M_{maxlag}$) in modern ecoregions, compared across biogeographic realms. Color coding as in Fig. 1B.

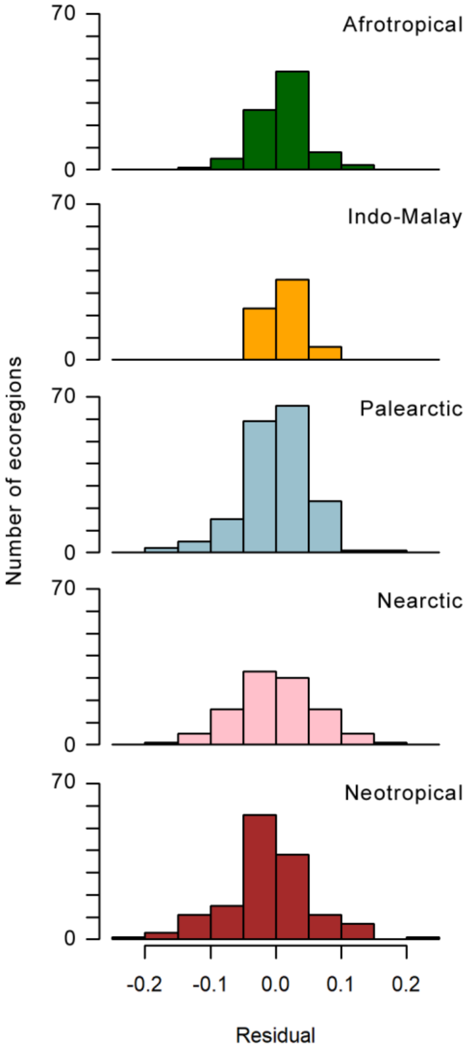

**Fig. S3:** Observed species temporal ranges (horizontal lines and dots [for single occurrences] based on locality midpoint ages) of leporid-like stem lagomorphs and leporids (orange), ochotonids (pink), artiodactyls (blue), and perissodactyls (dark blue) in North America from 43.5 to 1.5 Ma plotted against body masses (point estimates).

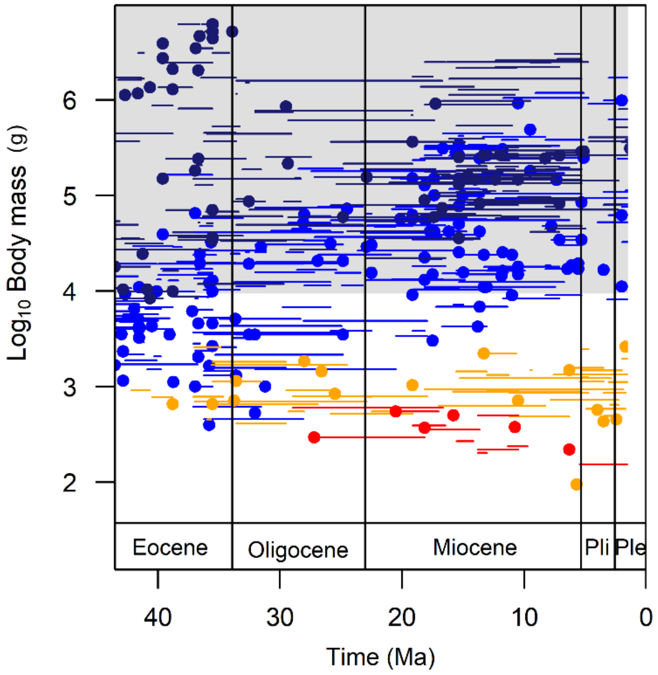

**Fig. S4:** Trajectories of body mass difference between minimum artiodactyl (A) or perissodactyl (B) and maximum lagomorph in North America from 43.5 to 1.5 Ma. Gray lines for 1,000 pseudo-replicates as in Fig. 3A; black line connects medians.

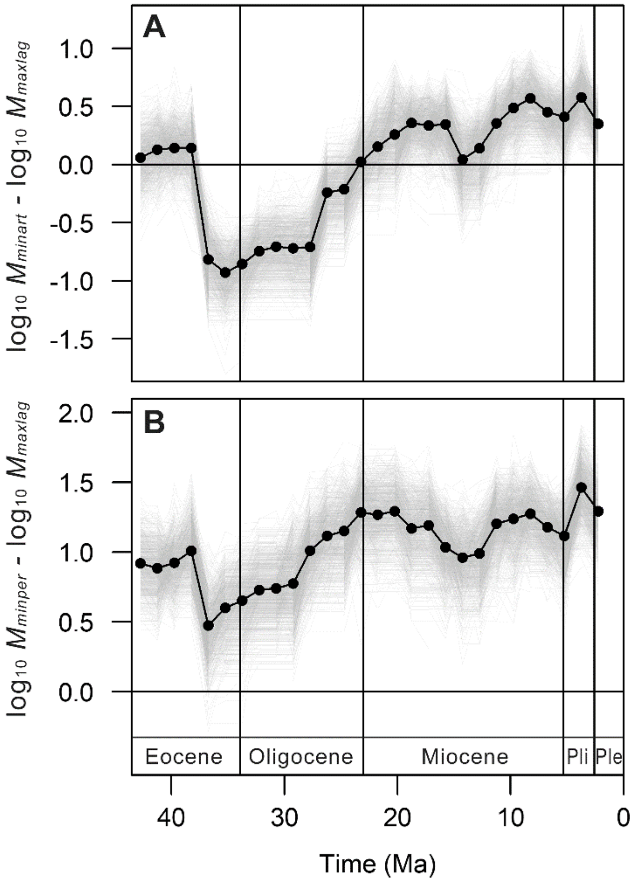

**Fig. S5:** Bivariate plot of maximum lagomorph and minimum artiodactyl body masses in 547 modern ecoregions. Color coding as in Fig. 1B. Asterisks represent body-mass combinations at the realm level.

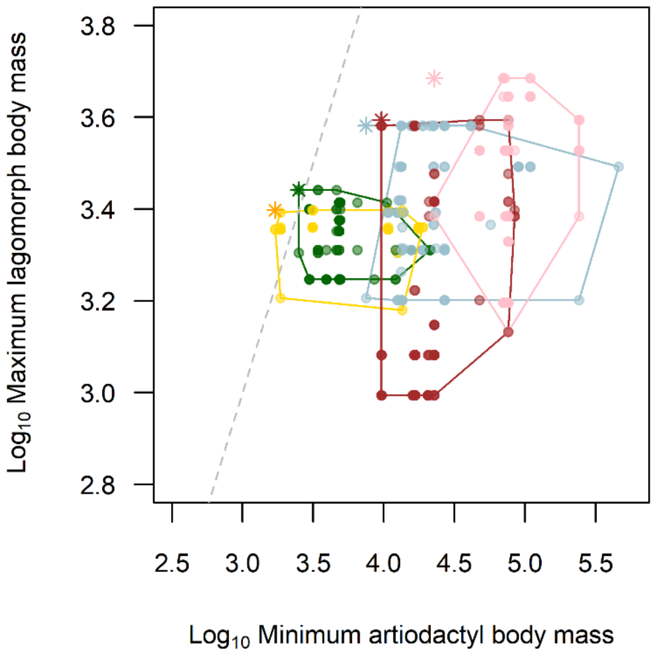

**Fig. S6.** Illustration of dental predictor variables used in lagomorph body-mass estimation (*Lepus townsendii* [MVZ 105670] as an example).

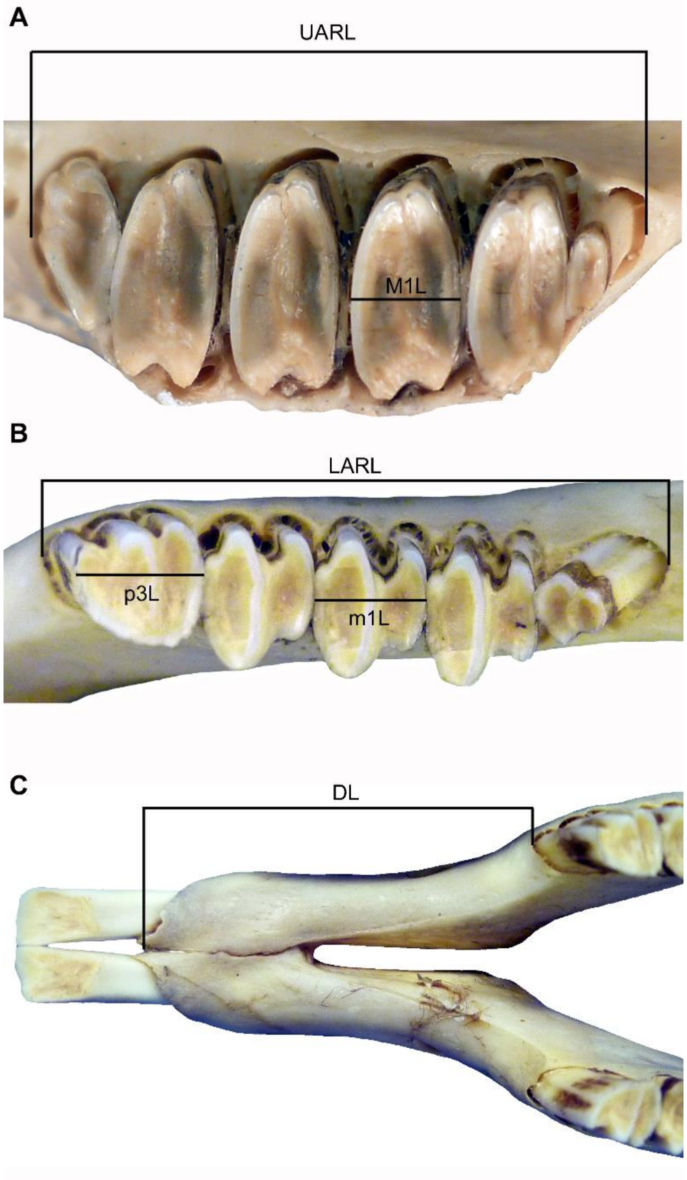

**Table S1.** Parameter estimates for lagomorph body-mass prediction models (in order of decreasing predictive accuracy as measured by $\overline{|\boldsymbol{D}|}$).

| Predictor | $\widehat{\lambda_{\mathrm{ML}}}$ | $\widehat{\beta_0}$ | $\widehat{\beta_1}$ | $\widehat{\sigma_{\beta_0}}$ | $\widehat{\sigma_{\beta_1}}$ | $\widehat{\sigma_y{}^2}$ | $\widehat{\sigma_x{}^2}$ | $R^2$ | $\overline{\lvert D \rvert}$ |
|---|---|---|---|---|---|---|---|---|---|
| TL* | 0.00 | -3.40 | 2.47 | 0.18 | 0.08 | 0.000 | 0.038 | 0.992 | NA |
| DL | 0.00 | 0.22 | 2.38 | 0.09 | 0.08 | 0.002 | 0.046 | 0.969 | 0.075 |
| M1L | 0.98 | 2.02 | 3.14 | 0.10 | 0.32 | 0.019 | 0.021 | 0.828 | 0.087 |
| m1L | 1.00 | 1.62 | 3.34 | 0.12 | 0.31 | 0.018 | 0.028 | 0.870 | 0.089 |
| LARL** | 0.96 | -0.57 | 3.09 | 0.22 | 0.22 | 0.008 | 0.019 | 0.905 | 0.089 |
| UARL** | 1.00 | -0.81 | 3.34 | 0.26 | 0.26 | 0.011 | 0.024 | 0.889 | 0.090 |
| p3L | 0.64 | 1.92 | 2.44 | 0.08 | 0.20 | 0.010 | 0.015 | 0.840 | 0.111 |

*For extant species only. **These predictors were ultimately not used in body-mass estimation of fossil species. Parameters: $\lambda$, Pagel's $\lambda$; $\beta_0$, intercept; $\beta_1$, slope; $\sigma_{\beta_0}$, standard error in intercept; $\sigma_{\beta_1}$, standard error in slope; $\sigma_y{}^2$, response variance constant; $\sigma_x{}^2$, predictor variance constant. See text for predictor abbreviations and Fig. S6 for illustration. $\overline{|D|}$ denotes mean absolute deviation of predictions from observed body masses.

**Table S2.** Parameter estimates for ungulate body-mass prediction models (in order of decreasing predictive accuracy).

| Predictor | $\widehat{\lambda_{\mathrm{ML}}}$ | $\widehat{\beta_0}$ | $\widehat{\beta_1}$ | $\widehat{\sigma_{\beta_0}}$ | $\widehat{\sigma_{\beta_1}}$ | $\widehat{\sigma_y}$ | $\overline{\lvert D \rvert}$ |
|---|---|---|---|---|---|---|---|
| LMRL | 0.70 | -0.41 | 3.03 | 0.17 | 0.08 | 0.184 | 0.119 |
| m2L | 0.81 | 1.09 | 3.01 | 0.15 | 0.09 | 0.209 | 0.127 |
| m1L | 0.77 | 1.17 | 3.10 | 0.15 | 0.09 | 0.211 | 0.134 |
| M2L | 0.79 | 1.19 | 2.91 | 0.17 | 0.10 | 0.243 | 0.149 |
| m1W* | 0.85 | 2.03 | 2.89 | 0.15 | 0.10 | 0.249 | 0.158 |
| M2W* | 0.77 | 1.26 | 3.06 | 0.15 | 0.10 | 0.221 | 0.164 |
| m2W* | 0.83 | 1.93 | 2.91 | 0.17 | 0.11 | 0.269 | 0.173 |
| m3W* | 0.86 | 1.99 | 2.94 | 0.17 | 0.11 | 0.276 | 0.179 |
| m3L | 0.87 | 1.15 | 2.82 | 0.17 | 0.09 | 0.248 | 0.195 |

*These predictors were ultimately not used in body-mass estimation of fossil species. See Table S1 for parameter notations and SI text for predictor abbreviations.

**Table S3.** GLS model parameters for population density *D* and ‘basal’ metabolic rate *R* against body mass in extant leporids and ungulates (non-‘suoid’ artiodactyls and perissodactyls).

| Response | Group | $N$ | $\widehat{\lambda_{ML}}$ | Intercept (SE) | Slope (SE) |
|---|---|---|---|---|---|
| $\log_{10} D$ | Leporidae | 19 | 0* | 7.06 (1.71) | -1.68 (0.53) |
| $\log_{10} D$ | Artiodactyla + Perissodactyla | 116 | 0.30 | 2.25 (0.65) | -0.42 (0.12) |
| $\log_{10} R$ | Leporidae + Artiodactyla† | 16 | 0* | 0.81 (0.13) | 0.67 (0.03) |

*Equivalent to ordinary least-squares regression models. †No data were available for perissodactyls with population density data.

**Literature Cited in Supplementary Information**


Alroy, J. 1998. Cope's rule and the dynamics of body mass evolution in North American fossil mammals. Science 280:731–734.

Bartoszek, K. 2019. R package "GLSME", ver. 1.0.5.

Bininda-Emonds, O. R., M. Cardillo, K. E. Jones, R. D. MacPhee, R. M. Beck, R. Grenyer, S. A. Price, R. A. Vos, J. L. Gittleman, and A. Purvis. 2007. The delayed rise of present-day mammals. Nature 446:507–512.

Bininda-Emonds, O. R. P., M. Cardillo, K. E. Jones, R. D. E. MacPhee, R. M. D. Beck, R. Grenyer, S. A. Price, R. A. Vos, J. L. Gittleman, and A. Purvis. 2008. Corrigendum: The delayed rise of present-day mammals. Nature 456:1038.

Capellini, I., C. Venditti, and R. A. Barton. 2010. Phylogeny and metabolic scaling in mammals. Ecology 91:2783–2793.

Dawson, M. R. 2008. Lagomorpha. Pp. 293–310 *in* C. M. Janis, G. F. Gunnell, and M. D. Uhen, eds. Evolution of Tertiary Mammals of North America, Vol. 2: small mammals, xenarthrans, and marine mammals. Cambridge University Press, Cambridge.

Garland, T., and A. R. Ives. 2000. Using the past to predict the present: confidence intervals for regression equations in phylogenetic comparative methods. The American Naturalist 155:346–364.

Ge, D., Z. Wen, L. Xia, Z. Zhang, M. Erbajeva, C. Huang, and Q. Yang. 2013. Evolutionary history of lagomorphs in response to global environmental change. PLoS One 8.

Hansen, T. F., and K. Bartoszek. 2012. Interpreting the evolutionary regression: the interplay between observational and biological errors in phylogenetic comparative studies. Systematic Biology 61:413–425.

IUCN. 2013. The IUCN Red List of Threatened Species.

Kelly, T. S. 2000. A new Hemphillian (Late Miocene) mammalian fauna from Hoye Canyon, west central Nevada. Contribution in Science (Natural History Museum of Los Angeles County) 481:1–21.

Legendre, S. 1986. Analysis of mammalian communities from the late Eocene and Oligocene of southern France. Palaeovertebrata 16:191–212.

Maddison, W. P., and D. R. Maddison. 2018. Mesquite: a modular system for evolutionary analysis.

Martins, E. P., and T. F. Hansen. 1997. Phylogenies and the comparative method: a general approach to incorporating phylogenetic information into the analysis of interspecific data. The American Naturalist 149:646–667.

Mendoza, M., C. M. Janis, and P. Palmqvist. 2006. Estimating the body mass of extinct ungulates: a study on the use of multiple regression. Journal of Zoology 270:90–101.

Moncunill-Solé, B., J. Quintana, X. Jordana, P. Engelbrektsson, and M. Köhler. 2015. The weight of fossil leporids and ochotonids: body mass estimation models for the order Lagomorpha. Journal of Zoology 295:269–278.

Pagel, M. 1999. Inferring the historical patterns of biological evolution. Nature 401:877–884.

Paradis, E., and K. Schliep. 2019. ape 5.0: an environment for modern phylogenetics and evolutionary analyses in R. Bioinformatics 35:526–528.

Pinheiro, J., D. Bates, S. DebRoy, D. Sarkar, S. Heisterkamp, B. Van Willigen, and R. Maintainer. 2017. Package ‘nlme.’ Linear and nonlinear mixed effects models, version 3.

R Development Core Team. 2018. R: a language and environment for statistical computing. R Foundation for Statistical Computing, Vienna.

Revell, L. J. 2010. Phylogenetic signal and linear regression on species data. Methods in Ecology and Evolution 1:319–329.

Rose, K. D., V. B. DeLeon, P. Missiaen, R. S. Rana, A. Sahni, L. Singh, and T. Smith. 2008. Early Eocene lagomorph (Mammalia) from Western India and the early diversification of Lagomorpha. Proceedings of the Royal Society B: Biological Sciences 275:1203–1208.

Smith, R. J. 1993. Logarithmic transformation bias in allometry. American Journal of Physical Anthropology 90:215–228.

Tomiya, S., and L. K. Miller. 2021. Data from: Why aren't rabbits and hares larger? Dryad Digital Repository, doi: https://doi.org/10.5061/dryad.ns1rn8ps3.

White, J. A. 1988. The Archaeolaginae (Mammalia, Lagomorpha) of North America, excluding *Archaeolagus* and *Panolax*. Journal of Vertebrate Paleontology 7:425–450.

White, J. A. 1991. North American Leporinae (Mammalia: Lagomorpha) from late Miocene (Clarendonian) to latest Pliocene (Blancan). Journal of Vertebrate Paleontology 11:67–89.
